# After Treatment Decrease of Bone Marrow Tregs and Outcome in Younger Patients with Newly Diagnosed Acute Myeloid Leukemia

**DOI:** 10.1155/2020/2134647

**Published:** 2020-11-04

**Authors:** Mario Delia, Paola Carluccio, Anna Mestice, Roberta Frappampina, Francesco Albano, Giorgina Specchia, Pellegrino Musto

**Affiliations:** ^1^Hematology and Bone Marrow Transplantation Unit-Azienda Ospedaliero-Universitaria Consorziale Policlinico, Department of Emergency and Organ Transplantation-University of Bari “Aldo Moro”, Bari, Italy; ^2^University of Bari “Aldo Moro”, Bari, Italy

## Abstract

An emerging body of evidence demonstrates that defects in antileukemic effector cells in patients with acute myeloid leukemia (AML) can contribute to the development and/or persistence of the disease. In particular, immune suppressive regulatory T cells (Tregs) may contribute to this defective antileukemic immune response, being recruited by bone marrow leukemic cells to evade immune surveillance. We evaluated Tregs (CD4+/CD45RA-/CD25^high^/CD127^low^), performing multiparametric flow cytometry on freshly collected bone marrow aspirate (BMA), in addition to the usual molecular and cytogenetic work-up in newly diagnosed AML patients to look for any correlation between Tregs and the overall response rate (ORR). We studied 39 AML younger patients (<65 years), all treated with standard induction chemotherapy. ORR (complete remission (CR)+CR with incomplete hematologic recovery (CRi)) was documented in 21 out of 39 patients (54%); two partial responder patients were also recorded. Apart from the expected impact of the molecular-cytogenetic group (*p* = 0.03) and the NPM mutation (*p* = 0.05), diagnostic BMA Tregs did not show any correlation with ORR. However, although BMA Tregs did not differ in the study population after treatment, their counts significantly decreased in responder patients (*p* = 0.039), while no difference was documented in nonresponder ones. This suggested that the removal of Treg cells is able to evoke and enhance anti-AML immune response. However, the role of BMA Tregs in mediating immune system-AML interactions in the diagnostic and posttreatment phase should be confirmed in a greater number of patients.

## 1. Introduction

In recent years, considerable progress has been made in deciphering the molecular and genetic heterogeneity of acute myeloid leukemia (AML) [1] and in defining new diagnostic and prognostic biomarkers [2]. In particular, having an impact on the clinical outcome, the therapeutic strategy largely depends on the European LeukemiaNet genetic risk stratification [3].

Moreover, a growing number of recurring genetic changes have been recognized in the new World Health Organization (WHO) classification of AML [4]. It appears that the prognostic relevance of integrated genetic profiling [5] is crucial in the diagnostic phase and seems to explain the clinical complexity of AMLs which inevitably differ from each other. Consequently, novel therapies, targeting some of the known genetic lesions, are now amply appealing [6]. However, in spite of these new targeted drugs, the outcome of patients affected by AML remains dismal paving the way for emergent players.

In AML patients, the bone marrow (BM) aspirate, studied through a genomic and epigenomic-based approach [7], is part of the altered BM-AML-immune environment [8, 9]. The AML microenvironment may be analyzed to explore its potential ability to contribute to the disease onset and outcome since it is immunosuppressive and antiapoptotic by itself thus favoring the immune escape and survival of malignant hematopoietic cells [10].

Given the complex interactions between AML cells and the many components of their BM-environment [11–13], we should expect additional diagnostic and/or prognostic roles for bone marrow aspirate T regulatory cells (BMA Tregs), which previous studies have emphasized [10, 14, 15]. However, in solid tumors, the role of Tregs seems to be firmly associated with tumor escape from immunosurveillance and consequently a worse outcome [16, 17], whereas in the AML setting, their action is still matter of debate [18]. In fact, apart from selected lymphomas in which tumor infiltrating Treg cell density seems to be associated with a better outcome [19], there are conflicting results in terms of a worse [20–22] or better prognosis [23] in AML patients. Moreover, the possible role of Tregs was indistinctly studied analyzing BM [23], peripheral blood (PB) [20, 21, 24, 25] and BMA plus PB [22, 26] at different time points (diagnosis [20–24, 26], and recovery phase posttreatment [23–25]) which made it difficult to reach firm conclusions.

Therefore, the aim of our study was to update our previous report [27] in an attempt to clarify the possible prognostic impact on the overall response rate (ORR) and outcome of the BMA Tregs, evaluated at the diagnostic phase and after treatment, in a prospective series for younger patients affected by AML.

## 2. Materials and Methods

### 2.1. Patients

We prospectively analyzed newly diagnosed AML patients (≤65 years) treated at our institution from March 2016 to May 2020. Patients gave written informed consent to the collection of personal data in accordance with the Declaration of Helsinki and Italian law.

All patients underwent induction chemotherapy (i.e., “3 + 7”) with cytarabine 100 mg/m^2^, intravenously, on days 1 to 7 and an anthracycline (daunorubicin 60 mg/m^2^ on days 1 to 3 or mitoxantrone 10 mg/m^2^ on days 1 to 3) and midostaurin 50 mg twice daily, in case of FLT3 mutation (ITD or D835) on days 8-21 (since January 2018). Thereafter, responders underwent consolidation chemotherapy with cytarabine at higher doses (up to 2 cycles) and midostaurin 50 mg twice daily in case of FLT3 mutation (ITD or D835) on days 8-21 (since January 2018), and nonresponders underwent salvage chemotherapy (FLAG-Ida for all patients) as a bridge to allotransplantation. The AML response was evaluated according to the ELN-2017 [3] limiting the evaluation of the quality of response to the morphologic complete response (CR). At the time of the evaluation of response, BMA-T cell population (CD3+,CD4+; CD8+; Tregs) together with B (CD19+) and natural killer (NK) cells count was repeated for comparison with diagnostic samples. A subanalysis of BMA-Treg count reduction in midostaurin-treated FLT3-mutated AML and low-risk patients was also performed.

### 2.2. Flow Cytometry

To determine the percentage and the absolute count of CD3 and CD4 T cell subsets, 50 *μ*l of whole marrow blood was stained with CD45 PerCP-Cy™5.5, CD3 FITC, CD4 PE-Cy7™, CD8 APC-Cy7, CD16 and CD56 PE, and CD19 APC monoclonal antibodies (MoAbs) (BD Multitest 6-color TBNK) in a calibrated number of fluorescent beads (Truecount, BD Parmingen). For Treg identification, 100 *μ*l of marrow blood was incubated with a lyophilised pellet of CD45RA FITC, CD25 PE, CD127 PerCP-Cy™ 5.5, HLA-DR PE-CY™7, CD39 APC, and CD4 APC-H7 MoAbs (BD Pharmingen). Samples were processed according to the manufacturer's guidelines and acquired on a DB FACS Canto II Flow Cytometer. The absolute number (cells/*μ*L) of positive cells was calculated by comparing cellular events to bead events using BD FACSCanto clinical software (version 3).

### 2.3. Treg Populations

BM-Tregs we found were

- CD4+/CD127^low^/CD25^high^
- CD4+/CD45RA-/CD127^low^/CD25^high^ (study population)
- CD4+/CD45RA-/CD127^low^/CD25^high^/DR+/39+

There is not a generally accepted method to define CD4 + CD25^high^ Tregs using flow cytometry, and FoxP3 is considered to be one of the most specific markers of Tregs [28]. Nevertheless, Tregs consistently express lower levels of IL-7R (CD127) than the majority of other CD4+ T cells, and the CD127 expression is inversely correlated with FoxP3 levels in Tregs [29, 30]. Thus, CD127 might be a suitable alternative to FoxP3 in identifying Tregs. As a result, our Treg population has been defined as CD4+/CD45RA-/CD25^high^/CD127^low^.

### 2.4. Statistical Analysis

We were interested in studying Tregs in responder patients. Accordingly, assuming that 70 ± 15% of AML patients were responder, along with a confidence coefficient (1 − *α*) = 0.95 [confidence level =95%] and a confidence interval = 0.7 ± 0.15, respectively, we needed 36 AML patients to treat.

The Mann–Whitney rank sum test was used to compare absolute cell counts, while chi-square or Fisher's exact test (2-tailed) was performed to compare proportions. The comparison of the diagnostic BMA (dBMA) population with the posttreatment one was performed with paired *t*-test or Willcoxon signed rank test, as appropriate. The variables analyzed for a correlation with ORR were age, white blood cells (WBC), integrated molecular-cytogenetic risk, the NPM mutation, the FLT3 ITD or D835 mutation, NPM^mut^FLT3^wt^Normal Karyotype, BMA Tregs, and de novo vs secondary AML. Covariates in the multivariate logistic regression models were chosen by stepwise-with-backward elimination variable selection procedures. The discriminatory power of the dBMA Treg value to predict response was assessed by estimating the area under the ROC curve (AUC). The optimal cutoff was determined by maximizing both sensitivity and specificity, computed at the optimal cutoff, as reported along with the 95% confidence intervals. The variables analyzed for a correlation with the dBMA Treg median value were age, WBC, integrated molecular-cytogenetic risk, the NPM mutation, the FLT3 ITD or D835 mutation, and de novo vs secondary AML. The overall survival curves were plotted with the Kaplan-Meier method and compared by the log-rank test, not censoring patients at allotransplantation. Significance was defined as a *p* value of ≤0.05.

## 3. Results

### 3.1. Study Population

Patient characteristics are summarized in Table 1. The study included 39 AML patients (18 males and 21 females, median age 56 years, range 19-65). According to cytogenetic-molecular risk stratification [31], 3 (8%), 26 (66%), and 10 (26%) patients were assigned to favorable, intermediate, and adverse prognosis groups, respectively. Molecular evaluation (i.e., NPM, FLT3, and CEBPA) was performed in all cases. NPM1 (A or B mutation) and FLT3 mutations (ITD or D835) were positive in 8 (20%) and 9 (23%) patients, respectively. There were no CEBPA positive cases. Median values of white blood cells (WBC) and of dBMA Tregs were 17000/*μ*L and14/*μ*L, respectively.

### 3.2. CD4-Total Event Count

The median number of CD4-total events counted by flow cytometry analysis in diagnostic and after treatment phase was 86779 (range (r): 11242-176351) and 73948 (r: 17163-244274), respectively.

### 3.3. dBMA Cell Count

**Figure 1:**
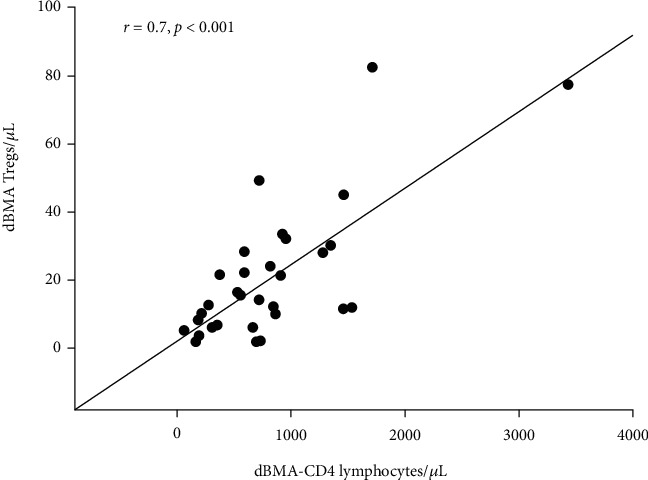
Diagnostic bone marrow aspirate (dBMA) CD4-lymphocyte correlation with dBMA Tregs (*r* = 0.70, *p* < 0.001).

dBMA CD4 lymphocytes showed a correlation with dBMA Tregs (*r* = 0.7, *p* < 0.001, Figure 1).

**Figure 2:**
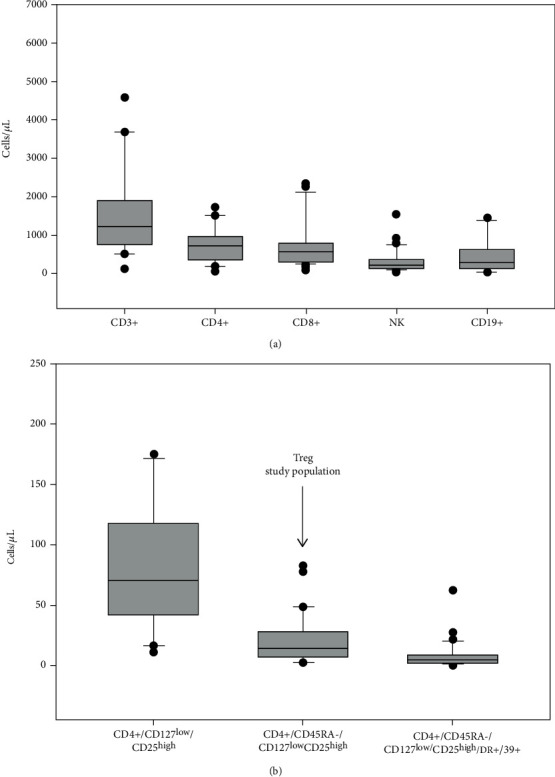
Box plots showing values of diagnostic bone marrow aspirate (dBMA) CD3 (median value, mv 1064/*μ*l),-CD4 (mv 595/*μ*L),-CD8 (mv 561/*μ*L),-natural killer (NK, mv 225/*μ*L), and -B lymphocytes (mv 296/*μ*/*μ*L) (a) and three Treg populations: CD4+/CD127^low^/CD25^high^ (mv, 60/*μ*L), CD4+/CD127^low^/CD25^high^ (mv, 14/*μ*L), and CD4+/CD45RA-/CD127^low^/CD25^high^/DR+/39+ (mv, 4/*μ*L); arrow indicates the Treg study population (b).

Median diagnostic CD3, CD4, CD8, NK, and B-cell values were 1064/*μ*L (*r*: 115-6600), 595/*μ*L (*r*: 64-3432), 561/*μ*L (*r*: 64-2324), 225/*μ*L (*r*: 16-1536), and 296/*μ*L (*r*:0-5280), respectively (Figure 2(a)). Median dBMA Tregs CD4+/CD127^low^/CD25^high^, CD4+/CD45RA-/CD127^low^/CD25^high^, and CD4+/CD45RA-/CD127^low^/CD25^high^/DR+/39+ were 60/*μ*L (*r*: 4-210), 14/*μ*L (*r*: 2-82), and 4/*μ*L (*r*: 0-62), respectively (Figure 2(b)).

10% (60 out of 595 CD4/uL) of CD4 + Tcells were Tregs CD4+/CD127^low^/CD25^high^.

### 3.4. After Treatment BMA-Cell Count Reduction

**Figure 3:**
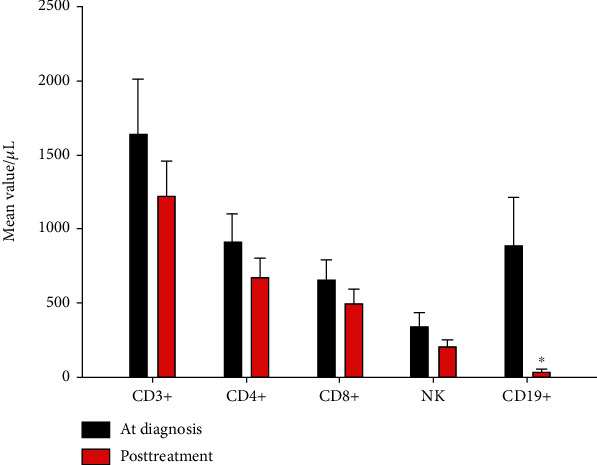
Bone marrow aspirate- (BMA-) CD3, -CD4, -CD8, -NK, and -B lymphocyte count reduction after antineoplastic treatment, data shown as mean ± SE. BMA-CD3, -CD4, -CD8, -NK, and -B count reduction from 1638/*μ*L (diagnostic phase: black bar) to 1222/*μ*L (after treatment phase: red bar) (*p* = ns), 909/*μ*L to 672/*μ*L (*p* = ns), 657/*μ*L to 496/*μ*L, (*p* = ns), 341/*μ*L to 208/*μ*L (*p* = ns), and 883/*μ*L to 34/*μ*L (^∗^*p* < 0.001, Wilcoxon signed rank test), respectively.

The after treatment-BMA-CD3, -CD4, -CD8, -NK, and -B-population count decreased from the mean value of 1638/*μ*L to 1222/*μ*L (*p* = ns), 909/*μ*L to 672/*μ*L (*p* = ns), 657/*μ*L to 496/*μ*L (*p* = ns), 341/*μ*L to 208/*μ*L (*p* = ns), and 883/*μ*L to 34/*μ*L (*p* < 0.001, Wilcoxon signed rank test), respectively (Figure 3).

**Figure 4:**
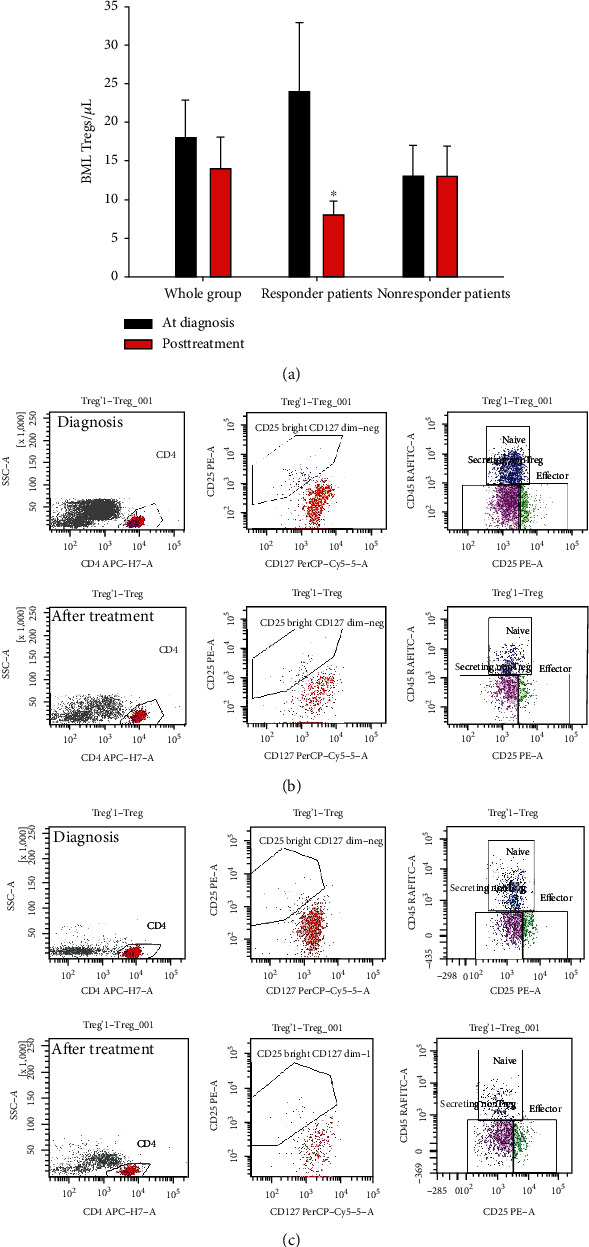
Bone marrow aspirate- (BMA-) Treg count reduction after antineoplastic treatment, data shown as mean ± SE. BMA-Treg reduction in the whole group, in responder and nonresponder patients from 18/*μ*L (diagnostic phase: black bar) to 14/*μ*L (after treatment phase: red bar) (*p* = ns), 24/*μ*L to 8/*μ*L (^∗^*p* = 0.039, Wilcoxon signed rank test), and 10/*μ*L to 10/*μ*L (*p* = ns), respectively (a). Flow cytometry plot in a responder (b) and nonresponder patient (c) diagnostic and after treatment evaluations.

The after treatment-BMA-Treg count did not statistically decrease within the whole group (mean dBMA Tregs 18/*μ*L vs after treatment Tregs14/*μ*L, *p* = ns (whole group bars, Figure 4). Analyzing the difference between patients achieving response from those who did not, BMA Tregs significantly decreased after treatment in responder patients (24/*μ*L vs 8/*μ*L, *p* = 0.039, Wilcoxon signed rank test, responder patients bars-Figure 4), while no difference was found in nonresponder patients (10/*μ*L vs 10/*μ*L, *p* = ns, nonresponder patients bars, Figure 4).

The three low-risk patients clearly showed a BMA-Treg count reduction from the value of 30/*μ*L to 23/*μ*L, 28/*μ*L to 12/*μ*L, and 10/*μ*L to 0/*μ*L, respectively.

**Figure 5:**
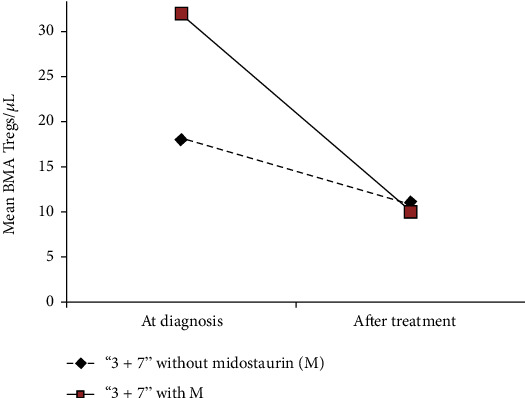
Bone marrow aspirate- (BMA-) Treg count modification after “3 + 7” treatment with (4 patients) or without midostaurin (35 patients). Mean BMA-Treg value decreased from 32/*μ*L to 10/*μ*L and from 18/*μ*L to 11/*μ*L in “3 + 7” treated patients with or without midostaurin, respectively.

Mean BMA-Treg value decreased from 32/*μ*L to 10/*μ*L and from 18/*μ*L to 11/*μ*L in “3 + 7” treated patients with or without midostaurin, respectively (Figure 5). In particular, the four midostaurin-treated patients (out of the nine ITD-mutated patients) showed a BMA-Treg trend from the value of 6/*μ*L to 5/*μ*L, 2/*μ*L to 6/*μ*L, 77/*μ*L to 14/*μ*L, and 45/*μ*L to 15/*μ*L, respectively.

### 3.5. Correlation between dBMA Tregs and AML-Related Prognostic Factors

Based on the dBMA Treg median value (14/*μ*L), the following did not show any correlation with dBMA Treg values ≤ 14/*μ*L: age, WBC, molecular-cytogenetics risk, the NPM mutation, the FLT3 ITD or D835 mutation, and the secondary AML (Table 2).

### 3.6. Factors Affecting ORR

**Figure 6:**
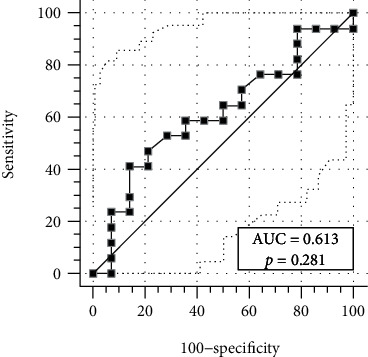
ROC curve: AUC analysis (AUC 0.61, *p* = 0.28), no optimal dBMA Treg cutoff value for predicting response to treatment.

ORR (CR+CR with incomplete hematologic recovery (CRi)) was documented in 21 out of 39 patients (54%). There were two partial responder patients. The factors affecting ORR were molecular-cytogenetic risk and NPM mutation both in univariate (*p* = 0.02 and *p* = 0.004) and in multivariate analysis (*p* = 0.03 and *p* = 0.05). Age, WBC, FLT3 mutation, dBMA Tregs, and de novo AML were not statistically associated with ORR (Table 3). ROC analysis did not detect any optimal dBMA Treg cutoff value for correlation with OR (AUC 0.61, *p* = 0.28, Figure 6).

### 3.7. Overall Survival (OS)

**Figure 7:**
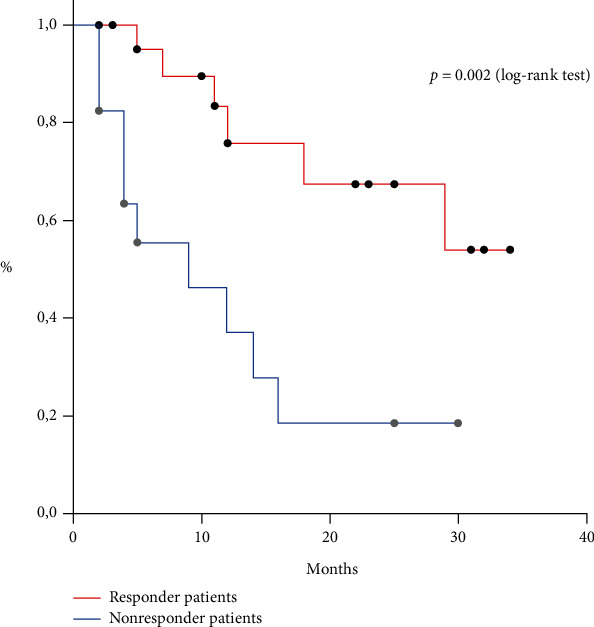
Median OS in responder and nonresponder patients (not reached vs 9 months, log-rank test, *p* = 0.002).

The whole group median OS was 18 months. The responder patient median OS was better than the nonresponder patient one (not reached vs 9 months, respectively, *p* = 0.002, log-rank test, Figure 7).

## 4. Discussion

Bearing in mind the complexity of the BM-AML microenvironment in which Tregs act, we have investigated their possible impact on response, adding a match-paired analysis of their absolute value differences before and after the antineoplastic treatment the study patients underwent.

It is well known that Tregs increase not only in the peripheral blood [20–22, 26] but also in the BM, where they seem to be higher and also more immunosuppressive [22, 26]. Confirming the dBMA Treg higher frequencies (10% of CD4 + T cells), our study population seemed suitable for studying Treg correlation with posttreatment outcome.

Interestingly, at the diagnostic phase, with regards to the BMA population other than the leukemic one, Williams et al. [32] did not report significant differences in the composition of the BMA Tregs by age, cytogenetic subgroup, or somatic myeloid-associated mutations. Accordingly, in our study population, dBMA Tregs neither seemed to correlate with clinical and molecular-cytogenetic risk category (Table 2) nor showed any ROC-threshold level predicting response (Figure 6).

On the other hand, the factors which have an impact on ORR are amply defined in an AML setting and have been confirmed in our analysis (Table 3). In particular, as expected, molecular and cytogenetic findings remain the major impacting variable both in univariate and in multivariate analysis, while no impact was demonstrated for dBMA Tregs. Therefore, our data might appear contradictory with the observations showing dBMA Tregs to be correlated with poor prognosis [22, 26], apart from their impact if studied in peripheral blood [20–23, 25, 26]. However, in our study population, we have analyzed the BMA Treg absolute values, instead of BM Treg frequencies, with the aim of studying the cytoreductive effect of antineoplastic treatment on BMA-T, -NK, and -B populations and the possible correlation with outcome.

It has already been shown [24] that treatment-induced lymphopenia is not a random process, and susceptibility to intensive chemotherapy differs between T cell subsets (i.e., CD4, CD8, and Tregs) and/or NK and B cells. Therefore, we were interested in deciphering the differential effects of treatment on the BM microenvironment with regard to Tregs and cells other than Tregs. While the only population that decreases after treatment is the B one (Figure 3), Tregs did not seem to reduce their absolute values in the whole group. Thus, our results seem to suggest that CD3+ T cells are less sensitive to intensive chemotherapy than B lymphocytes. Moreover, the preferential negative impact on B lymphocytes by chemotherapy in AML-treated patients obtaining CR has been already shown in studies that have investigated response to seasonal influenza vaccination as a surrogate for the robustness of the immune system [33]. In the same studies, frequencies of their T cell populations were similar to those seen in healthy controls [33].

Interestingly, BMA Tregs significantly decreased after treatment only in responder patients (Figure 4), thus miming, in the human setting, the in vitro evidence that Tregs and other T lymphocyte removal from the microenvironment lead to augmented immune responses to AML [15] and suggesting how dBMA Treg decrease matters irrespective of the higher pretherapeutic diagnostic values. Also noteworthy is that this treatment-correlated Treg reduction has been recently reported [34] in the context of the midostaurin-treated [35] AML patients with FLT3 mutation [36]. Although performed on peripheral blood, the study reported how midostaurin treatment significantly reduced the regulatory T cell population, suggesting an “off-target effect of this multikinase inhibitor on T cell signaling pathways” [34] as our subanalysis, though limited by a small number of patients and performed on BM, seemed to suggest (Figure 5). Moreover, it is known that AML cells secrete factors which inhibit T cell activation and proliferation [10, 15] and may directly drive Treg expansion [37].

## 5. Conclusions

It is widely recognized [18, 37, 38] that BMA-Treg values are higher in, and act at, the neoplastic AML site (i.e., bone marrow) primarily favoring leukemia growth. Additionally, in our study, unlike cytogenetics and molecular AML findings, dBMA Tregs do not correlate with response after antineoplastic treatment, confirming their role in sustaining the disease without being a trigger.

Nonetheless, it is well known that early lymphoid reconstitution after chemotherapy is associated with decreased risk of leukemia relapse [39], and these observations suggest that immunological events early after chemotherapy are clinically important too. In this context, the observed after-treatment BMA-Treg reduction in responder patients seems to suggest a new scenario. However, how the immunological status after treatment might be conditioned by preexisting disease-induced abnormalities or chemotherapy-induced defects needs to be further investigated.

## Figures and Tables

**Table 1:** Patients' characteristics.

	*n* = 39
Age	
Median value, range	56 (19-65)
Sex, *n* (%)	
Male	18 (46)
Female	21 (54)
AML FAB classification, *n* (%)	
M0	6 (15)
M1	7 (18)
M2	11 (28)
M4	9 (24)
M5	6 (15)
WBC/*μ*L	
Median value (range)	17000 (1400-281000)
Molecular/cytogenetics^@^, *n* (%)	
Good	3 (8)
Intermediate	26 (66)
Poor	10 (26)
NPM1 mutated, *n* (%)	8 (20)
FLT3 mutated, *n* (%)	
ITD	7 (18)
D835	2 (5)
dBMA Tregs	
Median value (range)	14 (2-82)
Secondary AML, *n* (%)	6 (15)
Overall response, *n* (%)	21 (54)

^@^according to ELN 2010 [ITD allelic ratio not performed]. AML: acute myeloid leukemia; ITD: internal tandem duplication; FAB: French-American-British classification; WBC: white blood cells; dBMA Tregs: diagnostic bone marrow aspirate T regulatory cells.

**Table 2:** Correlation between dBMA Tregs and AML-related prognostic factors.

	dBMA Tregs		
	≤14/*μ*L	>14/*μ*L	
	*n* = 20	*n* = 19	*p*
Age			ns^a^
Years, median value	55	56	
Range	(32-65)	(19-65)	
WBC			ns^a^
WBC/*μ*l, median value	12500	18400	
Range	(1900-281000)	(1400-138000)	
Molecular/cytogenetic group^@^*n*, %			ns^b^
Poor	6 (30)	4 (21)	
Intermediate	13 (65)	13 (68)	
Good	1 (5)	2 (11)	
NPM/FLT3 *n*,%			
NPM1^mut^	2 (10)	6 (32)	ns^c^
NPM1^wt^	18 (90)	13 (68)	
FLT3 ITD+ or D835+	6 (30)	3 (16)	ns^c^
FLT3^wt^	14 (70)	16 (84)	
De novo AML, *n* %			ns^c^
Yes	17 (85)	16 (84)	
No	3 (15)	3 (16)	

^@^according to ELN 2010 [ITD allelic ratio not performed]. WBC: white blood cells; dBMA Tregs: diagnostic bone marrow aspirate T regulatory cells. AML: acute myeloid leukemia; ^a^Mann–Whitney rank sum test, ^b^chi-square test, ^c^Fisher's exact test; ns: not statistically significant.

**Table 3:** Factors affecting overall response.

	Response, *n* = 39 patients		
	Yes	No.	
	*n* = 21 (54%)	*n* = 18 (46%)	*p*
Age			
Years, median value	52	56	ns^a^
Range	(19-65)	(35-65)	
WBC			
WBC/*μ*l, median value	4750	21290	ns^a^
Range	(1400-146000)	(1900-281000)	
Molecular/cytogenetic group^@^*n*, %			**0.02** ^**b**^ **; 0.03** ^**d**^
Poor	2 (10)	8 (44)	
Intermediate	16 (76)	10 (56)	
Good	3 (14)	0 (0)	
NPM/FLT3 *n*, %			
NPM1^mut^	8 (38)	0 (0)	**0.004** ^**c**^ **; 0.05** ^**d**^
NPM1^wt^	13 (62)	18 (100)	
FLT3 ITD+ or D835+	5 (24)	4 (22)	ns^c^
FLT3^wt^	16 (76)	14 (78)	
NPM1^mut^/FLT3^wt^/NK	3 (14)	0 (0)	ns^c^
No (NPM^mut^/FLT3^wt^/Nk)	18 (86)	18 (100)	
dBMA Tregs			ns^a^
Median value	21	12	
Range	(2-78)	(2-82)	
De novo AML *n*, %			ns^c^
Yes	20 (95)	13 (72)	
No	1 (5)	5 (28)	

^@^according to ELN 2010 [ITD allelic ratio not performed]. WBC: white blood cells; dBMA Tregs: diagnostic bone marrow aspirate T regulatory cells; NK: normal karyotype; AML: acute myeloid leukemia. ^a^Mann–Whitney rank sum test, ^b^chi-square test, ^c^Fisher's exact test, ^d^Multivariate stepwise-backward elimination procedure; Bold values are statistically significant (*p* < 0.05).

## Data Availability

The data used to support the findings of this study are included within the article.
